# Thyroid Cancer Staging: Historical Evolution and Analysis From Macrocarcinoma to Microcarcinoma

**DOI:** 10.7759/cureus.81972

**Published:** 2025-04-09

**Authors:** Carlos S Duque, Carlos E Builes-Montaño, Catalina Tobón-Ospina, Alejandro Velez Hoyos, Juan G Sánchez, Andres F Londoño, Miguel Agudelo, Julio A Valencia, Juan P Dueñas, Maria F Palacio, Natalia Sierra

**Affiliations:** 1 Department of Surgery, Clinica Intermedica, Medellin, COL; 2 Department of Internal Medicine, Hospital Pablo Tobón Uribe, Medellin, COL; 3 Department of Endocrinology, Universidad de Antioquia, School of Medicine, Medellin, COL; 4 Division of Endocrinology, Diabetes, and Metabolism, Clínica Somer, Rionegro, COL; 5 School of Health Sciences, Universidad Pontificia Bolivariana, Medellin, COL; 6 Department of Pathology, Hospital Pablo Tobón Uribe, Medellin, COL; 7 Department of Surgery, Clinica (Corporación de Estudios de la Salud) CES, Medellin, COL; 8 Department of Surgery, Hospital Pablo Tobón Uribe, Medellin, COL; 9 Department of Hepatology, Temple University Hospital, Newark, USA; 10 Department of Surgery, Clinica El Rosario El Tesoro, Medellin, COL; 11 Department of Surgery, Hospital Militar Central, Medellin, COL; 12 Department of General Medicine, Universidad Corporación de Estudios de la Salud (CES), Medellin, COL

**Keywords:** classification, prognosis, risk, staging, surgery, thyroid cancer, tnm

## Abstract

The classification of thyroid cancer diagnosis and treatment has evolved dramatically since the Union for International Cancer Control (UICC) published the first staging system in 1968. A careful review of the eight published editions of well-differentiated thyroid cancer (WDTC) staging by the UICC and the American Joint Committee on Cancer (AJCC) was performed. Each edition was analyzed to clearly understand which development published and accepted by specialists treating thyroid cancer justified considering a new updated edition. This study presents a comprehensive review of the remarkable evolution of thyroid cancer staging, highlighting the various changes in several areas throughout the years and editions. There were surprising changes within the eight publications: the tumor size was progressively reduced from 4 cm in the first AJCC volume to less than 1 cm in the seventh and eighth UICC and AJCC editions, classifying these small, WDTCs known up to now as “microcarcinomas.” Extrathyroidal extension was accepted after the third edition; this description certainly plays a key role in today’s decisions to manage this tumor as a prognostic factor. The age specification of 45 years prevailed for seven consecutive publications until it was raised to 55 years in the eighth thyroid cancer staging system. Without a doubt, this iconic change allowed physicians around the world to give their 45-year-old thyroid cancer patients a more encouraging panorama of the disease with the new classification.

Over the course of nearly 57 years, thyroid cancer staging has undergone remarkable changes, reaching a level of certainty that not only provides recommendations for safer treatments with less surgery and adjunctive measures but also improves survival rates and patient safety.

## Introduction and background

It is well known that thyroid cancer, especially that which fits in the category of well-differentiated thyroid cancer (WDTC), behaves differently with a much better prognosis if compared to other types of cancer that affect the human body. They represent a set of classification and staging challenges, owing to their biological and clinical characteristics. In recent decades, several alternative staging systems for thyroid cancer have been proposed by organizations or groups of physicians around the world, venturing to recommend new ways of staging, among them but not limited to: European Organization for Research and Treatment of Cancer (EORTC); Age, Grade, Extent, and Size (AGES); Mayo Clinic Metastasis, Age, Completeness of Resection, Local Invasion, and Tumor Size (MACIS); Age, Metastasis, Extent, and Size (AMES), etc. The Tumor, Node, and Metastasis (TNM) staging system, published by the Union for International Cancer Control (UICC) and the American Joint Committee on Cancer (AJCC), has been the dominant classification for the past 57 years [1-7]. It garnered consensus with minor adjustments and demonstrated that it fulfilled the needs of healthcare professionals and researchers. It also appears to offer the most comprehensive and reliable description of the oncological scenarios and their association with prognosis and survival.

The origins of cancer staging date back to 1958, when Pierre Denoix, a French physician at the Gustave Roussy Institute in Paris, developed a staging method for breast and laryngeal cancers. Subsequently, in 1968, the UICC published its first staging system, which included thyroid cancer among more than 20 malignant tumors affecting the human body. Nine years later, in 1977, the AJCC published its first Manual in Cancer Staging [6,7].

This article provides not only a unique, detailed description of the process, evolution, and changes in the thyroid cancer staging system over the last 57 years that have not been addressed before, but also delivers a series of undisclosed facts that can be considered for future TNM revisions if found appropriate [6-21].

## Review

Methods

All 16 editions of the TNM classification for thyroid cancer, along with several complementary articles, were carefully reviewed by all the authors. Among many elements, particular attention was paid to how the information was presented in the initial and subsequent editions; the progressive downsizing of the tumor (T) classification from one edition to the next; the description of lymph node involvement (N); patient characteristics such as age and sex; and the way explanatory graphics were aligned with the corresponding text.

In addition to these materials, a PubMed search was conducted using the keywords thyroid cancer, TNM, staging, classification, and history to supplement the gathered information. Selected guidelines from the American Thyroid Association (ATA) were also consulted and compared with the available data. Medullary and anaplastic thyroid cancers were excluded from the study.

This analysis primarily focused on key advances in thyroid cancer research that influenced revisions in subsequent TNM editions. The research protocol was submitted to the Ethical Research Committee of Hospital Pablo Tobón Uribe, which determined that ethical approval was not required, as this documentary research study did not involve patient data.

Results

Retrieving the information for the first four editions was a significant challenge, as it was primarily available as scanned pages from the UICC and PDF files of previous AJCC editions available online and through medical libraries. In order to verify that the scarce information for these initial editions was correct, not only were the two organizations contacted, but also some of the published literature at that time was reviewed, confirming the description encountered.

Discussion

General Information

The UICC's first TNM staging system for thyroid cancer was published in 1968, followed by the AJCC's initial cancer staging manual in 1977. Initially, both editions provided a concise overview of thyroid cancer that evolved as more knowledge was gained. In 1987, the two agencies began to publish their new editions at more or less the same time with similar information. There are, however, differences in how the information was presented. The AJCC included three basic thyroid anatomy diagrams in its first four editions; beginning with the fifth edition, two new diagrams were added showing a right and left neck with its anatomic planes. The AJCC eighth edition enhanced its illustrations by making them more graphic and easier to comprehend in relation to the pattern of the staging moment (e.g., depicting the invasion of the trachea). In addition, prominent U.S. physicians consulted from various medical specialties related to thyroid cancer were included. The result was a high-quality, up-to-date chapter that can serve not only as a staging guideline but also as an abbreviated chapter on diagnosing and treating the disease [4-23].

Although the UICC has maintained its simple format for all of its publications since 1982, a number of TNM atlases and supplementary materials have been published, complementing the published TNM information and providing superb color images for the latest editions. These certainly offered a new perspective and easy-to-understand illustrations. In addition, some reference information facts were detailed in their supplements [6-14,24-27].

There have been remarkable advances in the diagnosis and treatment of thyroid cancer during these eight editions of the TNM staging evolution. Not only have CT, MRI, and PET-CT technologies been introduced during this period, but the improved sensitivity of ultrasound has led to the accurate description of malignant nodules less than 1 cm in size, which are referred to as microcarcinomas. Thyroglobulin and thyroglobulin antibodies were introduced between the second and third editions of both organizations; these blood tests continue to play a vital role today in the follow-up of patients with thyroid cancer. The description of the Bethesda classification for thyroid nodules not only created a consensus followed by thyroid physicians worldwide but also ushered in a series of new advances in thyroid biology that led to distinct and ever-evolving molecular investigations [28-36].

**Figure 1:**
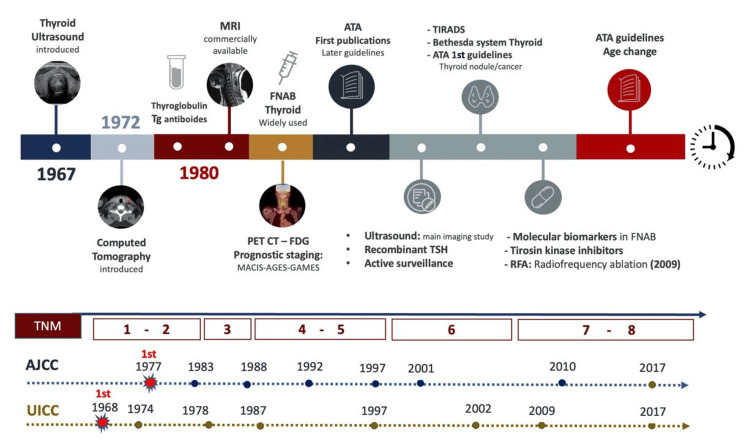
Historical developments in thyroid cancer diagnosis and treatments Developments and discoveries that took place throughout these eight UICC and AJCC editions of TNM thyroid cancer staging might have been considered in writing a new edition. UICC: Union for International Cancer Control, AJCC: American Joint Committee on Cancer, TNM: Tumor, Node, and Metastasis, ATA: American Thyroid Association, TIRADS: Thyroid Imaging Reporting and Data System, FNAB: fine-needle aspiration biopsy, MRI: magnetic resonance imaging, CT: computed tomography, PET CT – FDG: positron emission tomography with fluorodeoxyglucose, RFA: radiofrequency ablation, TSH: thyroid-stimulating hormone, Tg: thyroglobulin Image Credit: Catalina Tobón-Ospina (Author)

The observations and descriptions of thyroid cancer specialists in several fields of thyroid cancer treatment call for a reduction in the treatment of WDTCs less than 1 cm in size, offering eligible patients the option of active surveillance as an alternative to immediate surgery in a case-by-case decision. Another major step was the de-escalation of surgery by not recommending a total thyroidectomy for every patient with thyroid cancer with defined nodules measuring 1 to 4 cm in selected cases. All of these surgical changes were made possible by amazing advances in surgical technology (blood-vessel sealing devices, neuromonitoring, etc.) and patient care (Figure 1) [3,23-24,35-42].

Tumor Size (T)

**Figure 2:**
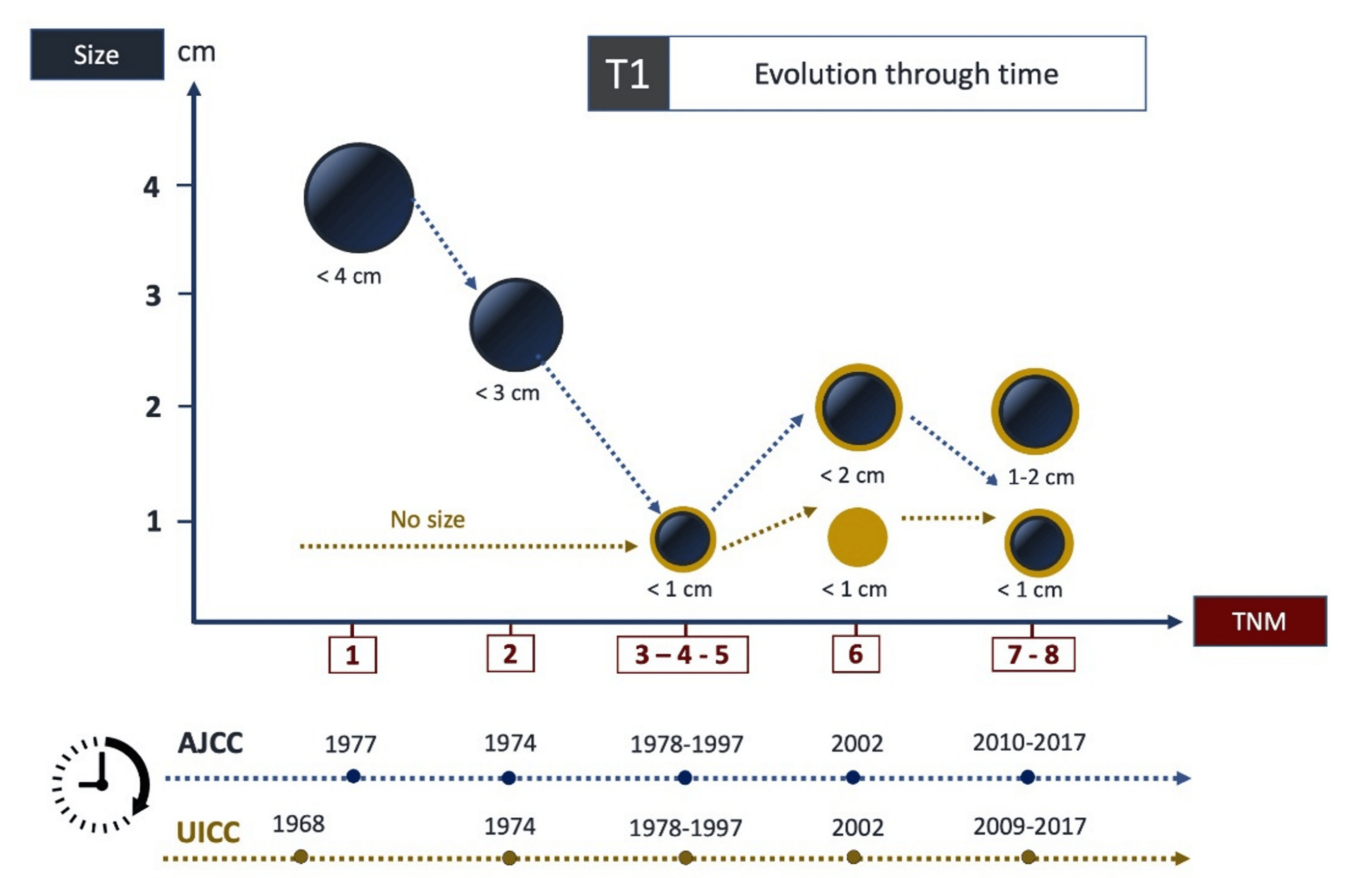
T1 size has reduced over the years and across editions The evolution and size reduction of T1 thyroid cancer over the years and across the eight editions of the AJCC and the UICC. TNM: Tumor, Node, and Metastasis, AJCC: American Joint Committee on Cancer, UICC: Union for International Cancer Control Image Credit: Catalina Tobón-Ospina (Author)

Tumor size (T) was addressed in the early publications of both organizations. These editions primarily relied on gross tumor findings, categorizing tumors up to the T3 stage if they extended beyond the gland limits. The UICC's first edition referred to a T1 as a “single tumor confined to the gland, no limitations of mobility or deformity,” with no reference to size. In contrast, the initial edition of the AJCC characterized a T1 thyroid cancer nodule as a T1a if it were less than 4 cm and a T1b if it was beyond the 4cm range at the time of diagnosis and treatment. In their subsequent edition, the T1 size was reduced to 3 cm, followed by a series of substantial modifications until they agreed with the UICC, establishing the 1 cm parameter in 1987. Today, a malignant nodule more than 4 cm would be classified as a T3 or T4, contingent on various tumor-related factors, including the extent of invasion into surrounding tissues, strap muscles, trachea, and recurrent laryngeal nerve, among others. For the seventh and eighth editions, a nodule measuring less than 2 cm was classified as a T1, with the notable distinction that a T1a was designated as a microcarcinoma if the nodule size was equal to or less than 1 cm and as a T1b if it measured between 1 and 2 cm (Figure 2) [6-24].

The T2 category has remained largely consistent over time, with minor modifications. The UICC referred to it as “multiple tumors or a single tumor with glandular deformity.” Both organizations described and agreed upon minimal changes to finally reach today's accepted range of 2 to 4 centimeters. The T3 category was assigned in the first editions of both agencies as a tumor extending beyond the gland or fixed to the surrounding tissues. This characterization underwent progressive modification, leading to its current interpretation. The contemporary understanding encompasses tumors not exceeding 4 cm or any malignant nodule exhibiting limited extrathyroidal extension. This revised definition is formally designated as T3a for tumors confined to the thyroid gland and T3b for those that extend beyond the thyroid capsule [4-21].

The T4 classification underwent a significant evolution, initially not being considered in the first and second editions of the UICC or the first edition of the AJCC. However, the UICC’s third edition introduced more objective criteria, such as gross invasion of surrounding tissues observed in preoperative images or by the surgeon, leading to the term “extrathyroidal extension.” After eight editions, the classification underwent a series of revisions, culminating in the subdivision of T4 into two distinct categories: T4a, which encompasses invasion to structures such as the recurrent laryngeal nerve and trachea, and T4b, which signifies a frank invasion of major neck vessels or the prevertebral fascia [4-21].

It is important to dwell a few moments on the term microcarcinoma, not only because it is part of the present and future vocabulary in WDTC, but also because the word itself has triggered many changes in WDTC management. It is based primarily on size, a nodule equal to or less than 1 cm, as there is no information on tumor biology at all [35-38]. This molecular aspect might be considered in future TNM editions on thyroid cancer, especially if health providers really want to make definitive considerations in risk stratification, prognosis, and survival. It will make a difference if an 8 mm WDTC lesion is, for example, BRAF positive or negative. It will mean an enormous distinction in suggesting a treatment decision if an aggressive pattern is found [4,35-39,41].

Lymphadenopathy (N) and Metastasis (M)

For the AJCC inaugural edition (1977), an N1 status was designated for any palpable nodes. The noteworthy subclassification was delineated as N1a if the nodes were on the same side, N1b if they were bilateral, and N1c if the nodes were bilateral or midline. Any fixed node was designated as N2, a classification that was excluded from subsequent AJCC editions. Conversely, the UICC characterized any “fixed nodules,” irrespective of their dimensions, as N3 in their first three editions. In the case of contralaterally or bilaterally involved lymph nodes, an N2 was categorized. A transitional period with minimal changes ensued, persisting until the fourth edition; at this point, only an N1 classification was considered. However, from the fourth to the sixth editions, the N1 status was applied to unilateral lymph nodes, and the presence of bilateral contralateral, midline, or even mediastinal (which, it should be noted, marked the first description of this anatomical region) nodes was referred to as an N2. The seventh and eighth editions introduced the N1 status as it is currently understood, delineating it into N1a if the nodes were loco-regional, referring to nearby lymph nodes, or N1b if they were found in the neck. The metastasis (M) element has remained unaltered throughout these eight editions [6-21].

While the most recent editions of the thyroid cancer staging classification accentuate the anatomical depiction of involved lymph nodes as a pivotal element, it is important to acknowledge the possibility of future revisions that may diverge from this trend. The advent of new pathological characterizations, not only related to present and newly described genetic mutations and tumor markers but also including the number and size of positive nodes, the presence of gross or minimal extranodal extension, and the involvement of perineural, vascular, or lymphatic channels, could bring about new classifications. In the contemporary context, these factors play a pivotal role in determining a patient's risk prognosis, determining whether he or she will require radioactive iodine treatment, and ascertaining the necessity of a complete thyroidectomy only if a hemithyroidectomy was performed [37-39,41,42].

Staging

Initially three UICC publications did not include a staging classification, and the AJCC initial edition did not contemplate it. However, this agency did include a staging sheet from the second through the seventh editions, distinguishing between patients under or over the 45-year range. The UICC initiated the incorporation of a staging group beginning with the fourth edition and has continued through the seventh edition, incorporating the 45-year age factor [6-21]

Age

**Figure 3:**
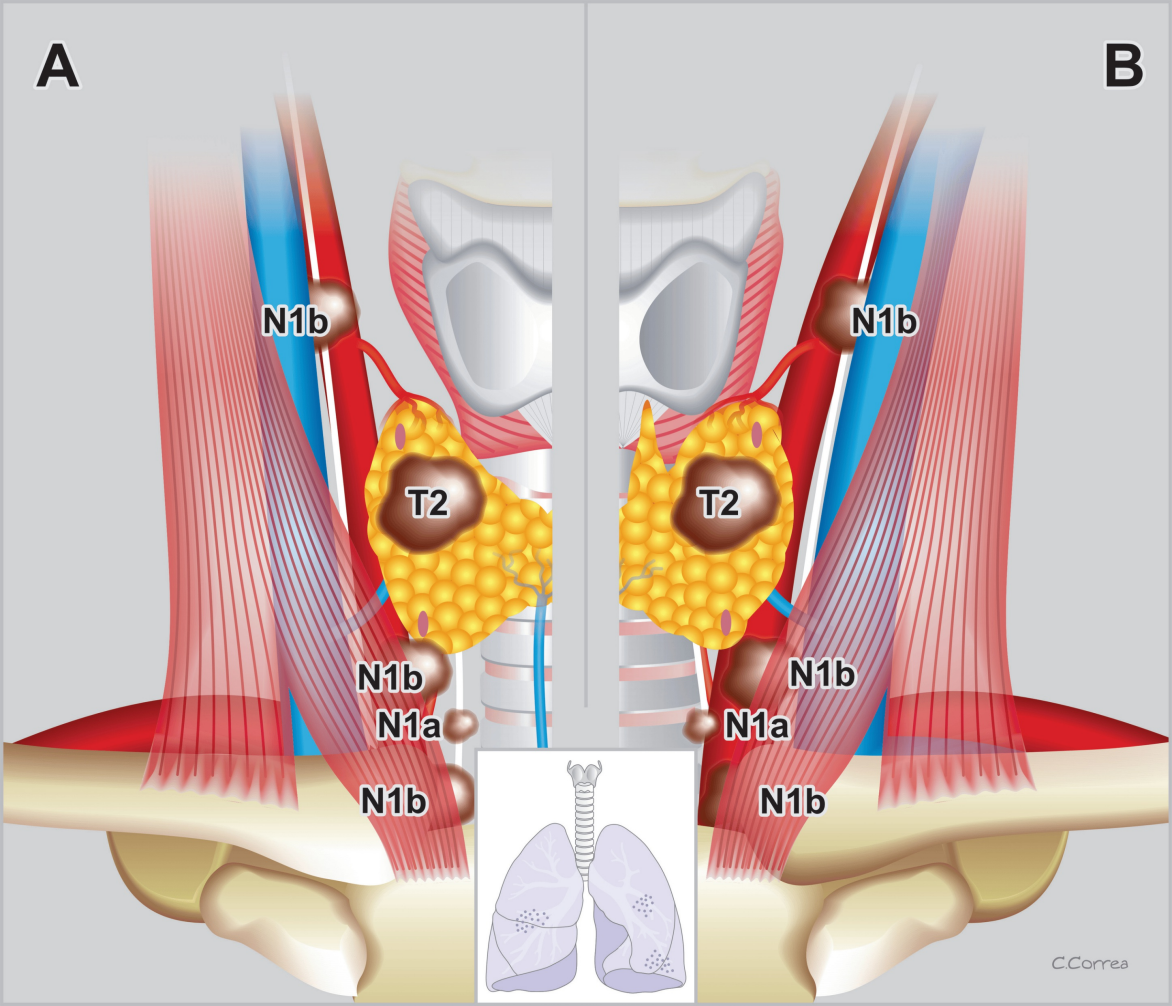
Stage classification according to age WDTC: staging differences in patients younger (A) or older (B) than 55. A: A 52-year-old female patient with a 2.6 cm, right thyroid lobe papillary cancer, with positive nodes in the neck and mediastinum, will be considered as Stage I. Even though this patient also debuts with lung metastases, it will be considered as Stage II. B: A 58-year-old female patient with a 2.6 cm, right thyroid lobe papillary cancer, with positive nodes in the neck and mediastinum, will be considered as Stage II. However, if this patient also debuts with lung metastases, it will be considered Stage IVb. WDTC: well-differentiated thyroid cancer Modified and adapted with permission from Duque (2022) [50]

The eighth edition of the UICC and AJCC introduced a significant advancement by incorporating age as a key staging factor. To the best of our knowledge, there are no similar references to another malignant tumor affecting the human body that considers the age element at the time of diagnosis as a prognostic and staging element. The 45-year age factor persisted for seven editions until a series of revisions were performed by several authors, prompting the discussion on the possibility of changing this number due to their observations of tumor behavior over the years [6-13,15-20]. This change finally happened in the eighth edition of both organizations; the age was upgraded to 55 years and accepted worldwide by the medical community [14,21]. This modification signified a crucial juncture, inciting worldwide revisions of multiple patients’ data. Medical practitioners sought to “reclassify” their patients, offering them novel information and renewed hope in the face of their tumor findings at the time of diagnosis and surgery (Figure 3) [43-49].

Sex

WDTC cases primarily affect women; however, it is interesting that none of these publications explicitly addressed the sex difference factor. The assumption was implicit, or “assumed,” that the information would apply to both sexes. The sole mention of the sex factor was depicted in the fifth edition of the AJCC and the inaugural edition of the UICC Illustrated Atlas. The images depicted a male face, accompanied by sketches of the neck levels. However, this perspective might be influenced by the historical utilization of these illustrations for delineating lymph node involvement and dissemination in other head and neck cancers, such as the larynx and pharynx. In contrast, the AJCC’s sixth edition clearly depicts a female face [51-53].

Future

The future of thyroid cancer diagnosis and treatment looks promising. Remarkable developments in all sorts of thyroid cancer-related fields have been described, giving physicians around the world advanced tools, such as new molecular testing exams that help to clarify, in some cases, a diagnosis, ruling out malignancy, and avoiding unnecessary surgeries. Besides present and newly developed molecular studies, they would provide patients with a more precise risk assessment and prognosis. Additionally, the surgical treatment of thyroid cancer will definitely adopt a more conservative approach, moving from a total thyroidectomy to a partial/hemithyroidectomy procedure, reducing complications such as bilateral laryngeal nerve damage or hypoparathyroidism, thus enhancing patient safety. Novel treatment alternatives, such as radiofrequency ablation, which has proven to be useful for treating benign thyroid nodules, are now beginning to be indicated for limited thyroid cancer, known up to now as microcarcinoma, and are now under investigation to approve their use, eliminating the tumor and avoiding surgery, especially potentially competing with the active surveillance protocol [54-57]. Even though thyroid cancer has great overall survival rates, there are still a few patients with the advanced or metastatic disease and even anaplastic carcinoma who will benefit from newly and constantly developed chemotherapy/biological or so-called systemic therapies that will offer affected patients a novel treatment opportunity not seen before [57]. Above all, the Internet has not only enhanced communication among researchers worldwide, facilitating knowledge sharing, but also emerging technologies, particularly artificial intelligence (AI), are poised to play a significant role in this field. AI will not only assist in analyzing radiologic images and pathology slides but might also offer solutions to complex algorithms. Furthermore, suppose a physician who treats thyroid cancer inputs different data from a patient into an AI program, among those referred to in the text (age, sex, size and histology of tumor, number of lymph nodes affected, extrathyroidal extension, vascular or lymphatic invasion, etc.). In that case, that program will be able to classify more clearly the general risk of tumor recurrence and the final prognosis of the patient's situation. Future editions may also consider other facts that have not been discussed or even mentioned in prior publications. For example, the pediatric population diagnosed with WDTC, on whom the less than 55-year age factor does not apply, even though some of those patients younger than 18 years old might be diagnosed initially with an advanced disease [58-62].

## Conclusions

The thyroid cancer staging system has undergone significant changes, including the reduction of the size of a nodule from a T1 registered as a 4 cm malignant nodule to a 1 cm or even smaller thyroid cancer, to be finally called a microcarcinoma. The universal acknowledgment of the term extrathyroidal extension, whether gross or minimal, plays a crucial role in thyroid cancer risk assessment and the treatment decision. Upgrading the age factor from 45 to 55 years at the time of diagnosis and treatment was a paramount recommendation, allowing the re-staging of a patient's initial classification in numerous instances. Finally, molecular studies are now an important tool in the diagnostic process of a thyroid nodule. However, they may play a fundamental role in future thyroid cancer staging, as the test might become useful to select treatment for early disease and advanced disease.
